# Potential Therapeutic Strategies for Lung and Breast Cancers through Understanding the Anti-Angiogenesis Resistance Mechanisms

**DOI:** 10.3390/ijms21020565

**Published:** 2020-01-15

**Authors:** Wafaa S. Ramadan, Dana M. Zaher, Alaa M. Altaie, Iman M. Talaat, Adel Elmoselhi

**Affiliations:** 1 College of Medicine, University of Sharjah, Sharjah 27272, UAE; wafaa.s.ramadan@hotmail.com (W.S.R.); U17105878@sharjah.ac.ae (D.M.Z.); U17200755@sharjah.ac.ae (A.M.A.); amoselhi@sharjah.ac.ae (A.E.); 2 Sharjah Institute for Medical Research, University of Sharjah, Sharjah 27272, UAE; 3 Pathology Department, Faculty of Medicine, Alexandria University, 21526 Alexandria, Egypt; 4 Department of Physiology, Michigan State University, East Lansing, MI 48824, USA

**Keywords:** angiogenesis, resistance, angiogenesis inhibitors, breast cancer, lung cancer

## Abstract

Breast and lung cancers are among the top cancer types in terms of incidence and mortality burden worldwide. One of the challenges in the treatment of breast and lung cancers is their resistance to administered drugs, as observed with angiogenesis inhibitors. Based on clinical and pre-clinical findings, these two types of cancers have gained the ability to resist angiogenesis inhibitors through several mechanisms that rely on cellular and extracellular factors. This resistance is mediated through angiogenesis-independent vascularization, and it is related to cancer cells and their microenvironment. The mechanisms that cancer cells utilize include metabolic symbiosis and invasion, and they also take advantage of neighboring cells like macrophages, endothelial cells, myeloid and adipose cells. Overcoming resistance is of great interest, and researchers are investigating possible strategies to enhance sensitivity towards angiogenesis inhibitors. These strategies involved targeting multiple players in angiogenesis, epigenetics, hypoxia, cellular metabolism and the immune system. This review aims to discuss the mechanisms of resistance to angiogenesis inhibitors and to highlight recently developed approaches to overcome this resistance.

## 1. Introduction

Cancer is one of the most common causes of human death globally [1]. Currently, there is a great demand for anticancer therapies with high specificity and low toxicity. Recently, most anticancer research has focused on the mechanisms of anticancer drugs [2], in particular anti-angiogenic drugs [3]. The research of angiogenesis and its role in tumorigenesis was established and termed by Judah Folkman in early 1970 [4]. His research prompted the discovery of novel angiogenesis regulatory molecules and introduced the development of angiogenesis inhibitors [5].

Within the human body, angiogenesis, which is the formation of new blood vessels from preexisting ones, is regulated by two types of molecules/factors: the pro-angiogenic molecules as vascular endothelial growth factors (VEGFs) and the anti-angiogenic molecules as thrombospondins (TSP) and angiostatin [6,7,8,9,10,11]. In a tumor lesion, the angiogenic balance is shifted toward being pro-angiogenic [12]; this shifting is known as the angiogenic switch [13,14]. Tumor hypoxia is regarded as the principle pathological cause that mediates this switch because it enhances the expression of pro-angiogenic factors [15]. Neoplastic cells and some host cells like macrophages also release several pro-angiogenic factors, such as collagenase and plasminogen activators, that cause disruptions of the basement membrane of the surrounding vasculature and trigger angiogenesis [14]. In addition, these molecules act as chemotactic factors for endothelial cells [16] and enhance the differentiation of the circulating bone marrow progenitor cells into endothelial cells [17]. As the series continues, a new basement membrane formation is observed, and pericytes are recruited to support the new blood vessel [18]. Interestingly, angiogenesis facilitates the spread of the tumor cells and, hence, mediate metastasis [19].

VEGF is a sub-family of growth factors consisting of five members, VEGF A, VEGF B, VEGF C, VEGF D, and VEGF E, and placental growth factor (PGF). The secreted VEGF acts by binding to one of the three tyrosine kinase VEGF receptors (VEGFR) [20]. Then, the downstream signal is transduced through the PI3K/AKT, MAPK and RAS/RAF/MEK /ERK signaling pathways [21].

VEGFs play a crucial role in different conditions of health and disease. The critical behaviors of VGEFs in wound healing, embryonic development, and lymph-angiogenesis have been widely investigated and studied. Angiogenesis is regarded as an important step in wound healing, especially when hypoxia is established after injuries. This stimulus (hypoxia) upregulates the expression of angiogenic cytokines such as VEGF-A, which is an essential factor for vascular formation, proliferation, survival, and, hence, recovery from ischemic injury through nutritional and oxygen supply [22]. Studies on wound closure have revealed a strong correlation between granulation tissue formation and VEGF-A [23,24,25,26,27,28,29]. This could be explained by its effect on fibroblast behavior and ultimately collagen production, as well as its arrangement during the wound healing process [30]. Along the same line, embryogenesis is also related to VEGF-A expression [31,32]. In the early stages of embryogenesis, the condensation of endothelial cells into blood vessels during the vasculogenesis process [33] tends to create the yolk sac vasculature, and this occurs outside the embryo. Later on, vasculogenesis occurs within the embryo proper to give rise the dorsal aorta. This endothelial cell differentiation and condensation is mainly dependent on VEGF-A, and the abolishing of VEGF-A expression in mice has been shown to result in early embryonic death [31,32]. In lymph-angiogenesis, similar to hemangiogenesis, the growth of lymphatic endothelial cells depends on the activation of VEGFR3 through VEGF-C induction [34,35]. The growth of lymphatic vessels is guided by the migration of the tip cells and the formation of filopodia (cellular protrusions) with the proliferation of endothelial cells behind the tip cell, which allows for the elongation of the branch [34,36]. Data from different studies have indicated that the absence of VEGF-C fails to induce the delamination of the endothelial cells in the cardinal vein of a developing mouse embryo. Accordingly, the primary lymph sac and the lymphatic network are prohibited to be established and formed [37,38]. In the same manner, VEGF-C is also necessary for adult lymph-angiogenesis [39]. The constant signaling of VEGF-C is mandatory for the maintenance of lymphatic capillaries in some tissues. The knocking-out of VEGF-C in adult mice leads to the slow destruction of intestinal lacteals; on the other hand, the maintenance of lymphatic vessels in dermis is independent of a continuous supply of VEGF-C [40,41].

The majority of solid tumors such as breast cancer [42], small cell lung cancer (SCLC), and non-small cell lung cancer (NSCLC) overexpress VEGF-A, making it the dominant target for anti-angiogenic drugs [43,44]. The rationale of the anti-angiogenic compounds proposed by Folkman was to starve cancer cells and induce their dormancy [45]. It is noteworthy that the VEGF-A pathway blockade is characterized by an early and transient phase in which vessels assume normal shape and function [46]. This normalization is characterized by the rescue of the balance between inhibitors and inducers of angiogenesis, the reduction of leakage and interstitial pressure, the improvement of tumor perfusion and oxygenation, and drug delivery [43]. However, a meta-analysis of randomized clinical trials for using antiangiogenic tyrosine kinase inhibitors (TKIs) in the treatment of advanced NSCLC showed no improvement in the disease control rates and overall survival (OS) of patients [47]. Furthermore, the results of clinical trials demonstrated that the clinical benefits of using VEGF inhibitors as a therapy in lung cancer is limited, due to the development of drug resistance [48]. Adding to that, the clinical benefit of bevacizumab, an anti-VEGF-A monoclonal antibody, against breast cancer was unexpectedly limited and showed only an enhancement in the progression free survival (PFS), lacking the improvement in the OS [49]. In addition, adverse events were observed, including nephrotic syndrome and hypertensive encephalopathy. Eventually, the emergence of impedance towards angiogenic blockers came to be regarded as the main struggle in cancer treatment by the anti-angiogenesis strategy. In this review, the mechanisms of resistance to anti-angiogenic drugs, especially in lung and breast cancers, are discussed, and the recently developed approaches to overcome this resistance will be presented.

## 2. Principles of Anti-Angiogenic Therapy

Immature, abnormal, and highly permeable vessels are the most common features of intratumoral vasculature. For the treatment of solid tumors, the inhibition of angiogenesis is regarded as a potential strategy that depends on cutting off the blood supply. This leads to generalized hypoxia and necrosis. Some anti-angiogenic agents, like anti-VEGFs and anti-VEGFRs monoclonal antibodies, are highly specific towards their targets, while multiple tyrosine kinase inhibitors are nonspecific angiogenesis inhibitors [50]. The general hypothesis aims to induce morphological changes that ultimately drive intratumoral vascular shutdown and tumor necrosis. This necrosis usually happens in a severe form, but this typically occurs at the central part of the tumor while the peripheries still contain viable tumor cells [51]. These viable tumor cells receive their oxygen and nutritional supports from the surrounding normal blood vessels [52]. However, it has been suggested that the most potent vascular disrupting agents are unable to prevent tumor growth by shutting down the tumor’s blood supply [53]. The vascular change that occur during the early phases of anti-angiogenic drug exposure is called “vascular normalization” [54]. In addition, the time span between anti-angiogenic agents-induced vascular normalization and the subsequent vascular shutdown is called “normalization window.” Combretastatin, which is an example of vascular disrupting agents, exhibits a normalization window that starts as early as four hours after the delivery of this anti-angiogenic drug [54].

The de novo prevention of angiogenesis, followed by silent tumor death, is the most appealing anti-cancer strategy [55]. In breast cancer, hypoxia-inducible factor (HIF-1) α is regarded as a crucial regulator of angiogenesis. It has been established that HIF-1α is highly expressed in poorly differentiated breast cancer than in well differentiated breast cancer [56]. HIF-1α activates the expression of VEGF in breast cancer [57] and in NSCLC [58]. Furthermore, increased angiogenesis, which could be measured by VEGF expression or the density of blood vessels, is an independent negative prognostic factor in early breast cancer [59]. The addition of bevacizumab in the treatment of some advanced cancers like NSCLC, ovarian cancer, and cervical cancer improved PFS and OS [60]. However, in metastatic breast cancer, bevacizumab did not show any OS improvement [61]. Eventually, the controversy regarding the clinical benefits of this drug needs to be investigated further; in the meantime, the search for another effective drug is critically needed. Additional confliction has also been observed in neoadjuvant and adjuvant settings. For example, neoadjuvant trails showed an improved pathologic complete response rate (pCR), while adjuvant trials failed to show any effect on disease-free survival (DFS) or OS [62]. Further negative trial findings were also obtained with other anti-angiogenic drugs like sunitinib (oral multi TKI) [63] and sorafenib (an inhibitor of the RAF kinase family) [64] in advanced breast cancer [65,66]. By contrast, bevacizumab was the first anti-angiogenic drug approved for use in NSCLC treatment in 2006. After the success of bevacizumab, several antibodies and TKI molecules have been investigated [67]. Both bevacizumab and ramucirumab (a fully human monoclonal antibody (Immunoglobulin G1(IgG1) against VEGFR2) have been shown to improve OS when added to standard first and second line chemotherapy, respectively. Additional benefits may be further obtained by incorporating new adjuvant agents and novel treatment strategies upon the use of bevacizumab and ramucirumab. Such situations can be seen with the promising results of the combined utility of bevacizumab and erlotinib (epidermal growth factor receptor (EGFR)-TKI) for EGFR-mutant cancers, a combination which has been shown to lead to improvements in PFS for more than six months [68]. With the emergence of immunotherapy treatments in lung cancer, it is still to be investigated whether combined angiogenesis blockers and checkpoint inhibitors have more gains or not. Though these benefits have been small in OS, many other drugs have failed to improve OS in NSCLC patients. The modest improvements in OS seen with both bevacizumab and ramucirumab can be clinically meaningful for patients who have a significantly shortened lifespan [67].

## 3. Mechanisms of Resistance to Anti-Angiogenesis Therapy

**Figure 1:**
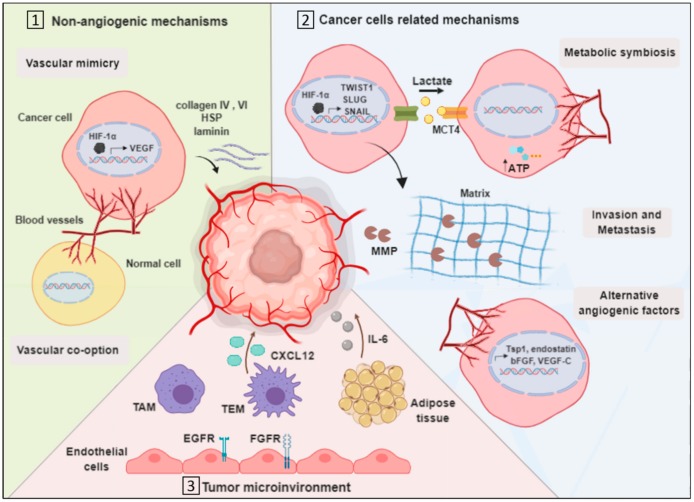
Mechanisms of resistance to anti-angiogenesis therapy. Tumor cells exhibit different mechanisms to resist anti-angiogenic therapy as (**1**) utilizing non-angiogenic mechanisms. In vascular mimicry, cancer cells form tubular structure supported by the secreted matrix proteins as collagen IV and VI, HSP, and laminin. In addition, in vascular co-option process, tumor cells take over existing vascular beds from adjacent normal tissues. (**2**) Cancer cell-related mechanisms to resist anti-angiogenic therapy include metabolic symbiosis, in which tumor cells generate adenosine triphosphate (ATP) by using lactate from other tumor cells that suffer from hypoxia, ultimately supporting vessels formation. Invasion contributes to resistance through the upregulation of extracellular matrix-related factors as MMP. The upregulated expression of alternative angiogenic factors is as well associated with resistance. (**3**) Tumor microenvironment-related mechanisms include the participation of the tumor stromal cells such as endothelial cells, TAM, TEM and myeloid and adipose cells in mediating resistance. Abbreviations: heparin sulfate proteoglycan, HSP; HIF-1α, Hypoxia inducible factor-1α; VEGF, vascular endothelial growth factor; MCT4, monocarboxylate transporter 4; ATP, adenosine triphosphate; MMP, matrix metalloproteinase; Tsp1, thrombospondin1; bFGF, basic fibroblast growth factor; TAM, tumor-associated macrophages; TEM, angiopoietin receptor (TIE2) expressing macrophages; EGFR, epidermal growth factor receptor; FGFR, fibroblast growth factor receptor; and IL-6, interleukin-6.

One of the challenges in the treatment of breast cancer is its resistance to administered drugs, as observed with angiogenesis inhibitors [69]. Based on clinical and pre-clinical findings, breast cancer has gained the ability to resist angiogenesis inhibitors through several mechanisms that rely on cancer cells and their microenvironment [70] (Figure 1).

### 3.1. Vascularization by Non-Angiogenic Mechanisms

#### 3.1.1. Vascular Mimicry

Some tumors have the capacity to form microvascular channels de novo, even in the absence of endothelial cells (EC), independent of angiogenesis. This process is called vascular mimicry (VM) and was introduced in 1999 by Maniotis et al. [71,72]. In this process, the tumor cells gain unusual endothelial-linked properties that make them able to form tubular structures that are supported by secreted matrix proteins such as collagen IV and VI, heparan sulfate proteoglycan (HSP) and laminin [73]. Several studies have revealed that VM is associated with aggressive breast and lung cancer types [71,74,75]. As with angiogenesis, growth factors and hypoxia-related factors, such as VEGF-A, vascular endothelial cadherin (VE-cadherin), platelet EC adhesion molecule (PECAM) and HIF-1 α, regulate VM [76]. When a tumor exhibits hypoxia, HIF-1 α is stabilized and promotes the transcription of angiogenesis-related genes such as the VEGF-A [20]. A recent in vitro study examined the effect of a VEGF-A blocker, bevacizumab, on the inhibition of VM in HCC1937 breast cancer cells. The study showed that bevacizumab failed to limit VM [77]. In addition, VM was shown to promote resistance to sunitinib [78]. Sunitinib is a receptor tyrosine kinase inhibitor that inhibits signaling by targeting all VEGFRs [79]. An in vivo study demonstrated that the administration of sunitinib to breast cancer model increases VM channel development and upregulates VM-associated proteins such as Twist1 [78]. Thus, VM appears to be associated with the angiogenesis signaling pathway, but it still has its unique mechanism that is not completely understood.

#### 3.1.2. Vascular Co-Option

The generation of tumor vasculature in the lungs, which are highly vascularized tissues, may occur through an alternative mechanism to angiogenesis called vessel or vascular co-option. In this angiogenesis-independent mechanism, tumor cells take over existing vascular beds from adjacent normal tissues, which means that there is no need to promote the formation of new blood vessels [73]. The co-option process in tumors occurs mainly when the tumor cells invade and grow predominantly in the perivascular environment of adjacent tissues. The other possible pathway happens in myofibroblast-rich tumors, in which tumor cells mediate and direct the translocation of the vasculature through mechanical forces that are mediated via active myofibroblasts [80,81]. The vital role of the vascular co-option mechanism to escape angiogenesis inhibitors has been demonstrated in both clinical and experimental studies [73]. Based on a preclinical lung metastasis model, it could be seen that treatment with the antiangiogenic drug sunitinib results in the induction of a switch from angiogenesis to vascular co-option, subsequently driving the acquired resistance to sunitinib [82]. Moreover, a study demonstrated that vascular co-option is highly implemented in the metastasis of breast cancer cells to the brain. Serpins, which is a class of plasminogen activator (PA) inhibitors, has been shown to be overexpressed in the brain metastases, facilitating their survival and vascular co-option capability [73]. The exact mechanisms and the specific players responsible for inducing cancer cells to use angiogenesis or vascular co-option for the formation of tumor vasculature are still not well understood. However, the biological properties of the tumor microenvironment seem to have a role in switching from angiogenesis to the vascular co-option to establish tumor vasculature [83].

### 3.2. Cancer Cell-Related Mechanisms

#### 3.2.1. Metabolic Symbiosis

Cancer energy metabolism has been of great interest to investigate and target due to its special characteristics compared to energy metabolism in normal cells. Normal tissues generate ATP through mitochondrial oxidative phosphorylation by converting glucose into water and carbon dioxide. This process happens in the presence of oxygen (aerobic conditions). However, under low oxygen conditions, normal tissues shift to lactic acid fermentation, metabolizing glucose into lactate. On the other hand, cancer cells, which are known for their uncontrolled growth rate, have a high demand for glucose to generate energy. Different from the metabolic behavior of normal cells, cancer cells utilize lactic acid fermentation, even with the presence of oxygen, a process which is called aerobic glycolysis (Warburg effect) [84]. Furthermore, cancer cells utilize the produced lactate for the production of building blocks such as fatty acids and amino acids to support their high proliferation rate [85]. Metabolic symbiosis, which is the generation of ATP by using lactate from tumor cells suffering from hypoxia, is another resistance-mediating mechanism [86]. Nintedanib, a drug that acts via the inhibition of angiogenesis-related kinases, has potent activity in the treatment of NSCLC and breast cancer [87,88]. Moreover, nintedanib has been assessed for resistance induction. Initially, the Py2T cells (resistant murine breast tumor cells) have been shown to respond to treatment with nintedanib. However, following three weeks of treatment, Py2T cells overcome treatment by upregulating lactate transporters such as monocarboxylate transporter 4 (MCT4), thus counteracting hypoxia [88]. Another study detected an overexpression of HIF-1α in NSCLC tissues [89]. Cancer cells exploit hypoxia to generate ATP, and they resist anti-angiogenic drugs by altering the expression of metabolism-related proteins. This phenomenon is considered a potential therapeutic target to restrain cancer cells’ sensitivity to angiogenesis inhibitors.

#### 3.2.2. Invasion

Invasion contributes to resistance through the upregulation of extracellular matrix (ECM)-related factors such as matrix metalloproteinase (MMP) [76,90,91]. MMPs contribute to the invasion process of the tumor cells through the degradation of the ECM [92]. This compensates for the induced hypoxia following treatment with angiogenesis inhibitors [19]. In addition, hypoxia has been reported to induce the expression of epithelial mesenchymal transition (EMT) genes (*TWIST1, SLUG* and *SNAIL*), thus promoting breast cancer’s invasion [93]. Moreover, the expression of these genes has been shown to be associated with poor prognosis in patients with NSCLC [94].

#### 3.2.3. Upregulation of Alternative Angiogenic Factors

The activation of compensatory pro-angiogenic pathways in response to anti-VEGF therapy has been investigated in lung cancer. The acquired resistance to aflibercept (VEGF-Trap), which inhibits VEGF-A and VEGF-B, in lung cancer cells was found to be associated with the upregulation of VEGF-C [95]. As VEGF-C is essential for the development of the lymphatic vasculature, it has also been found to be involved in tumor-induced angiogenesis by binding to its receptor, VEGFR3, which can be highly expressed in vascular endothelial cells of tumor-bearing tissues [96,97,98]. In addition, the up-regulation of Tsp1, endostatin, and basic fibroblast growth factor (bFGF) was also recorded in response to the treatment with angiogenesis inhibitors [99]. These findings demonstrated the capability of cancer cells to compensate for the inhibited angiogenic pathway.

### 3.3. Tumor Microenvironment-Related Mechanisms

Tumor cells initiation, growth, metastasis and angiogenesis are influenced by a population of cells that exist in the tumor microenvironment, and these cells are called tumor stromal cells [100]. This population consists of a variety of innate and adaptive inflammatory cells in addition to the endothelial cells and pericytes that comprise the angiogenic vasculature of the tumor [101]. In addition, fibroblasts and connective tissues are among the fundamental components of the tumor stroma. Alongside their participation in tumorigenesis, tumor stromal cells mediate resistance to anti-cancer therapies, including angiogenesis inhibitors [102].

#### 3.3.1. Endothelial Cells Mediated Resistance

A study resolved the mechanism by which breast cancer endothelial cells resist VEGF inhibition by the chemotherapeutic agent paclitaxel [103,104]. Resistance was mediated by the upregulation of ATP binding cassette transporters and multi-drug resistance proteins such as ABCB1 and ABCG2. In addition, Shojaei F et al. demonstrated the upregulation and activation of EGFR and FGF receptor (FGFR) in pericytes and endothelial cells in the surrounding stroma of the NSCLC xenograft model that acquired resistance to bevacizumab. These stromal signaling pathways promote VEGF-A-independent endothelial survival and increase the pericyte coverage of tumor vessels, which is important in tumor revascularization [105].

#### 3.3.2. Tumor-Associated Macrophages

Tumor-associated macrophages (TAM) are the other components of the tumor stromal cells with type-2 macrophage phenotype that exerts an immunosuppressive effect. A study revealed the importance of eotaxin and oncostatin M cytokines in recruiting TAMs to the site of breast cancer in a mouse model; hence, blocking these cytokines inhibited TAM infiltration and improved the tumor cells’ sensitivity to bevacizumab [106].

#### 3.3.3. TIE2 Expressing Macrophages

A subset of TAMs called angiopoietin receptor (TIE2) expressing macrophages (TEMs) are characterized by their expression of angiopoietin receptor TIE2 and their high pro-angiogenic activity. These properties are found to engage TEM in resistance development [107]. An in vivo study recently evaluated the efficacy of combretastatin A4 phosphate, an anti-angiogenic agent, in murine mammary tumors showed a limited response due the high levels of chemokine CXCL12 and the recruitment of TEMs to the site of the tumor [108].

#### 3.3.4. Myeloid and Adipose Cells

A recent study revealed myeloid and adipose cells’ capacity to induce resistance to bevacizumab in the breast cancer of obese patients [109]. The analysis of a phase 2 clinical trial elucidated a negative correlation between the sensitivity to anti-angiogenesis treatment and the levels of both interleukin-6 (IL-6) and FGF. A further in-depth investigation in murine breast cancer models supported the correlation, in which blocking the production of IL-6 from myeloid and adipose cells rendered cancer cells sensitive to bevacizumab treatment [109]. Moreover, the infiltration of myeloid cells to lung tumors has been associated with resistance to anti-VEGF-A drugs, as these cells are responsible for inducing tumor angiogenesis when the VEGF-A pathway is inhibited [110].

The contribution of the mentioned tumor stromal cells and many others as resistant to angiogenesis inhibitors makes them promising therapeutic targets. However, dysregulating the resistance-mediated pathways requires more adequate research to resolve the sophisticated interplay between tumor cells and their surroundings.

## 4. Strategies to Overcome Anti-Angiogenic Therapy Resistance

**Figure 2:**
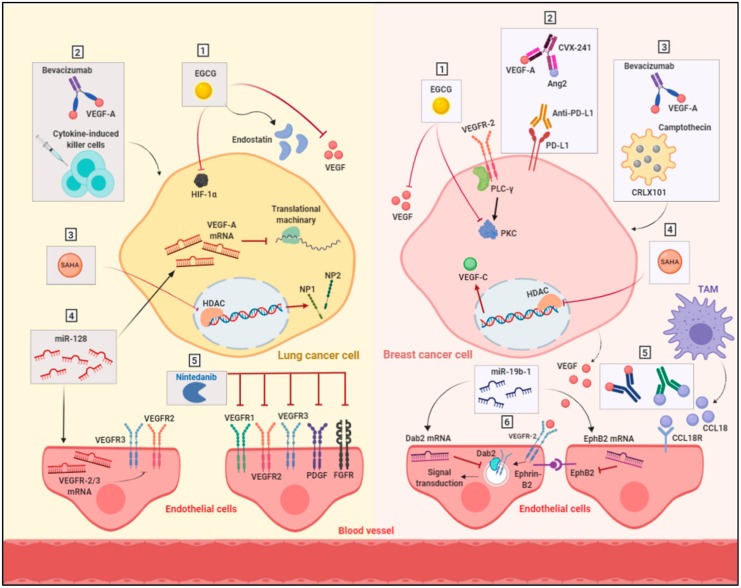
Strategies to overcome anti-angiogenic therapy resistance. Resistance to anti-angiogenic drugs in lung cancer (right) could be delayed by (**1**) the natural compound EGCG, which exerts its antiangiogenic effect by suppressing the expression of VEGF-A and HIF-1α and increasing the expression of angiogenesis inhibitor endostatin in cancer cells; (**2**) the combination of bevacizumab with immunotherapy, such as the adoptive transfer of cytokine-induced killer cells; (**3**) the inhibition of angiogenic VEGFR co-receptors NP1 and NP2 through histone deacetylase inhibitor SAHA; (**4**) the inhibition of the translation of VEGF-A mRNA in lung cancer cell and VEGFR-2 and VEGFR-3 mRNA in endothelial cells with microRNA-128; and (**5**) the use of the multi tyrosine kinase inhibitor nintedanib, which targets VEGFR1, VEGFR2, VEGFR3, PDGF, and FGFR. On the other hand, for breast cancer (right), the strategies to overcome anti-angiogenic therapy resistance include (**1**) the use of green tea extract EGCG to decrease the expression of VEGF transcription modulator PKC; (**2**) the combination of antiangiogenic antibody CVX-241 with the anti-programmed death-ligand 1 (anti-PD-L1) antibody; (**3**) the combination of bevacizumab with camptothecin which inhibits HIF-1α; (**4**) the use of epigenetic modulator SAHA to suppress VEGF-C expression; (**5**) the blocking of chemokine CCL18, which is secreted by TAM, and CCL18 receptor on endothelial cells; and (**6**) the inhibition of the signal transduction pathway of VEGFR2 in endothelial cells through the down-regulation of signaling regulators (Ephrin type-B receptor 2) EphB2 and Disabled homolog 2 (Dab2). Abbreviations: EGCG, epigallocatechin-3-O-gallate; VEGF, vascular endothelial growth factor; HIF-1α, hypoxia inducible factor-1α; SAHA, suberanilohydroxamic acid; HDAC, histone deacetylase; NP1, neuropilin1; NP2, neuropilin2; miR, microRNA; PDGF, platelet-derived growth factor receptor; FGFR, fibroblast growth factor receptor; Ang2, angiopoietin 2; PD-L1, programmed death-ligand 1; PLC-γ, phospholipase C-γ; PKC, protein kinase C; and TAM, tumor-associated macrophage.

There are some ongoing preclinical and clinical studies to investigate several strategies that may be used to overcome or delay resistance to anti-angiogenic drugs (Table 1) (Figure 2).

### 4.1. Targeting Multiple Angiogenic Proteins

Several anti-angiogenic drugs have been developed to target multiple regulators in angiogenesis pathways. Sorafenib, sunitinib and vandetanib are multi-targeted receptor tyrosine kinase inhibitors for angiogenesis; however, the outcome of the clinical trials for these inhibitors in NSCLC did not improve the overall survival of patients [111,112,113,114,115]. Similarly, several clinical trials for these multi-targeted angiogenic inhibitors showed a limited activity as a single agent in breast cancer patients [116].

Nintedanib is a multi-tyrosine kinase inhibitor that blocks the kinases domain of a variety of pro-angiogenic receptors. Nintedanib has been shown to be able to target VEGFRs (VEGFR1, VEGFR 2 and VEGFR3), platelet-derived growth factor receptor (PDGFa and b), FGFRs (1–4), fms-related tyrosine kinase 3 (FLT3), and SRC family kinases. The preclinical studies showed a promising inhibition in the tumor growth of NSCLC cells [117]. Later, the clinical trials demonstrated an improvement in overall survival of NSCLC patients and a better safety profile of nintedanib in comparison to other anti-angiogenic drugs. Nintedanib was approved by the European Medicine Agency as a second line of treatment in combination with docetaxel for treatment of NSCLC patients with histopathological characteristics of adenocarcinoma [118]. Additionally, there are some ongoing trials for the use of nintedanib alone or in combination with immunotherapeutic drugs for the treatment of metastatic NSCLC (Table 1).

### 4.2. Targeting Hypoxia

Hypoxia through HIF-1α factor highly contributes to the acquired resistance to angiogenic inhibitors. An in vivo study for a breast cancer model illustrated the impact of inhibiting HIF-1α via camptothecin (imbedded in a nanoparticle, CRLX101) in combination with bevacizumab. Breast tumors showed an improved response with a delayed recurrence [119]. In addition, an induced cancer stem cells population was retarded by bevacizumab treatment [119,120]. Clinically, a phase I trial in patients with advanced, refractory malignancies including breast and lung cancers demonstrated that the inhibition of angiogenic activity was achieved by a combination of bortezomib, an agent that inhibits HIF-1α, with a bevacizumab regimen [121].

### 4.3. Anti-Angiogenic Immunotherapy

As mentioned previously, inflammatory cells are key players in the resistance obstacle. In the last few years, several research studies have investigated the immunotherapeutic capacity alone or in combination to improve the cancer treatment [122]. In one of the performed in vivo studies, the expression of programmed death-ligand 1 (PD-L1) was found to be upregulated in the endothelium of breast tumors as a response to treatment with the mouse anti-VEGFR2 antibody DC101 [123]. Subsequently, this effect has promoted resistance [123]. Based on these findings, another group of researchers targeted VEGF-A and angiopoietin 2 in mouse breast cancer models by using multiple target anti-body in combination with the anti-PD-L1 antibody. This treatment protocol improved survival [124]. Interestingly, the combination of the adoptive transfer of cytokine-induced killer cells with bevacizumab synergistically inhibited the tumor growth in an in vivo lung adenocarcinoma model [125]. Furthermore, there is an ongoing phase I clinical trial for the combination of bevacizumab with anti-PD-1 nivolumab in patients with lung cancer to investigate the safety and tolerability of this combination regimen (NCT01454102). Another strategy was investigated based on the role of TAMs in mediating resistance through multiple pathways including the secretion of chemokines such as CCL18. An in vitro study further illustrated the role of CCL18 in promoting angiogenesis and tumor migration in breast cancer, as TAM’s involvement in mediating angiogenesis was diminished by blocking CCL18 and CCL18 receptors. This suggests CCL18 as a target to interrupt TAMs resistance-mediated pathways [126].

### 4.4. Targeting Epigenetic Regulators

Epigenetic changes involved DNA methylation, histone modification and microRNA expression are important regulators for VEGFs signaling. The status of histone modifications in a resistant lung tumor sample from VEGF-Trap-induced resistance in a Lewis lung carcinoma mouse model was changed in promoters of angiogenesis-related genes [127]. Furthermore, the inhibition of histone deacetylase (HDAC), which regulates histone deacetylation, by suberanilohydroxamic acid (SAHA) or trichostatin A (TSA) in lung cancer cell lines resulted in a dramatic decrease in the expression of VEGFR co-receptors neuropilin1 (NP1) and neuropilin2 (NP2). Additionally, the expression of VEGFR1 and VEGFR2 was also increased upon treatment with HDAC inhibitors [128]. On the other hand, in vitro studies in breast cancer cells have shown that the expression of VEGF-C is also reduced by the HDAC inhibitor SAHA in a dose-dependent manner [129]. Collectively, these studies suggest the important role of HDACs in regulating the expression of the VEGFs pathway.

MicroRNAs (miRs) are another level of regulation for gene expression of the VEGF–VEGFR pathways by inhibiting the translation of specific mRNAs. The MiR-200 family has been indicated to negatively regulate VEGFR1 and VEGFR2 expression in NSCLC cells [130,131]. In addition, miR-128 has been recorded to be down-regulated in NSCLC cells. Preclinical studies have shown that the ectopic overexpression of miR-128 in NSCLC cells results in the reduction of the expression of VEGF-C. This change could lead to a decrease in the expression of VEGF-A in NSCLC, VEGFR-2, and VEGFR-3 of endothelial cells. The suppression of angiogenesis in a NSCLC tumor xenograft model was observed following the restoration of miR-128 [132]. Therefore, the restoration of miR-128 or miR-200 could be a promising therapeutic strategy in lung cancer. In addition to that, miR-19b-1 overexpression in triple negative breast cancer cells showed a promising effect on arresting tumor growth and angiogenesis, possibly through the down-regulation of VEGFR2, (Ephrin type-B receptor 2) EphB2, and disabled homolog 2 (Dab2), all of which are involved in the internalization of VEGFR2 and the subsequent angiogenesis signaling transduction pathway [133]. However, further in vivo studies are required to assess the use of miR-19b-1 as a potential target in breast cancer—specifically in triple negative breast cancer.

### 4.5. Alternative Anti-Angiogenic Compounds

Natural compounds have recently been in the limelight for their safety and efficacy in the prevention and treatment of different types of malignancies [134]. Therefore, a great deal of research has been directed to study and discover natural agents with anti-angiogenic potential. Epigallocatechin-3-O-gallate (EGCG) is such a natural compound that is isolated from green tea and has been identified to act through multiple molecular mechanisms to inhibit angiogenesis in breast and lung cancers [135,136]. The anti-angiogenic effect of EGCG in NSCLC has been investigated in vitro and in vivo to suppress the expression of both HIF-1α and VEGF proteins, as well as to increase the level of angiogenesis inhibitor endostatin, which is produced from collagen degradation [135,137]. The effect of EGCG was also studied in a breast cancer xenograft model, in which EGCG reduced the density of tumor vessels, possibly by decreasing the expression of VEGF and protein kinase C, a VEGF transcription modulator [138]. Interestingly, EGCG underwent phase Ib and II clinical trials in breast cancer patients, and these suggested its potential chemopreventive effect on breast cancer risk among young women [139,140].

Other natural products have also been investigated to suppress angiogenesis in breast and lung cancers in preclinical models such as curcumin [141,142]. However, the results of phase I and II clinical studies of curcumin in other types of malignancies such as the skin, the liver and pancreatic cancer have not supported the preclinical observations due to the low stability and limited absorption of EGCG [143,144]. An interesting study was conducted by Wang L et al. in which they demonstrated the potential anti-cancer effect of using water-soluble liposomal curcumin on Lewis lung cancer in vitro and in vivo by suppressing angiogenesis [145]. This liposomal form of curcumin could overcome problems associated with previous clinical trials and may show a better improvement in the overall survival of cancer patients.

### 4.6. Modulation of Cell Metabolism

The metabolism of tumor cells and tumor ECs represents as a new target for anti-angiogenic therapy. Glycolysis is one of the metabolic pathways that ECs are highly relied on for providing sufficient energy for their motility and rapid division. The inhibition of glycolysis in ECs has emerged as a new approach in anti-cancer therapy. The activation of glycolysis in ECs carried out by the glycolytic activator phosphofruktokinase-2/fructose-2,6-bisphosphatase 3 (PFKFB3) is induced by VEGFR2 to promote the migration of ECs and stimulate angiogenesis [146]. The effect of PFKFB3 inhibition has been studied in tumor ECs, and this inhibition resulted in the tightening of the EC barrier by reducing the endocytosis of VE-cadherin and upregulating the expression of N-cadherin in pericytes, rendering them more quiescent and adhesive. These changes characteristically normalized the tumor vessels, thus improving blood perfusion and leading to a reduction in the intravasation and metastasis of cancer cells, as well as an increase in drug accessibility, which improves responses to chemotherapy [147]. This approach has been tested in NSCLC through the development of liposomes that carry plasmid-expressing short hairpin RNA against PFKFB3 and the chemotherapeutic drug docetaxel. This co-delivery system enhances the therapeutic efficacy of docetaxel through glycolysis inhibition in vivo by using a NSCLC xeno-graft nude mice model; this could be a new strategy for effective lung cancer treatment [148]. Indeed, the same approach could be tested for antiangiogenic therapies in lung cancer to overcome resistance to antiangiogenic drugs. On the other hand, the induction of metabolic symbiosis in hyperglycolytic cells has been reported in breast cancer models treated with anti-angiogenic multi-kinase inhibitors such as nintedanib. Nintedanib mediates metabolic symbiosis in cancer cells through the monocarboxylate transporter 4 (MCT4) protein, which is responsible for rapid lactate export in glycolytic cells. As discussed earlier, cancer cells rely on metabolic symbiosis to defend against the induced hypoxia by angiogenesis inhibitors. Fortunately, when nintedanib was combined with 3PO, a glycolysis inhibitor, resistance was relieved [88]. Targeting the key responsible enzymes for the elevated levels of metabolism in cancer cells has emerged as a potential therapeutic option. The inhibition of glycolysis as an adjuvant therapy to angiogenesis inhibitors could be a promising strategy to overcome resistance to antiangiogenic therapies.

## 5. Conclusions

The emergence of angiogenesis inhibitors in the treatment protocols of breast and lung cancers has been of great interest. However, their efficacy of treatment has not met expectations. The reason behind their limited anti-cancer activity has been investigated, and several resistance mechanisms have been found to be among the major factors. Breast and lung cancer cells have intrinsic capabilities to overcome hypoxia upon treatment with angiogenesis inhibitors. Among the most powerful mechanisms are vascular mimicry, metabolic symbiosis, metastasis and invasion. In addition, tumor stromal cells like endothelial cells, TAMs, TEMs, myeloid and adipose cells have been shown to have a major role in the development of resistance. The studies conducted to understand these mechanisms have suggested multiple potential targets to overcome resistance. Indeed, understanding the switch between angiogenesis and vascular co-option in lung cancer could result in the addition of a new strategy for overcoming resistance to anti-angiogenic drugs. Many studies are still in the in vivo stage, particularly in breast cancer, and they represent promising approaches. A further understanding of the angiogenesis inhibitors resistance mechanisms will reveal therapeutic targets and introduce new combination therapy protocols that will enhance angiogenesis inhibitors’ efficacy.

## Figures and Tables

**Table 1:** Preclinical and clinical studies for overcoming resistance to anti-angiogenic therapies.

Preclinical Studies			
Strategy	Targets		Main Conclusion
**Targeting multiple angiogenic proteins**	VEGFRs (1, 2 & 3), PDGFa & b, FGFRs (1–4), FLT3 and SRC family kinases via Nintedanib		Nintedanib showed promising inhibition in the tumor growth of NSCLC cells
**Targeting hypoxia**	HIF-1α via camptothecin		Combination of camptothecin with bevacizumab showed improved response of breast cancer cells and decreased the cancer stem cells population
**Anti-angiogenic immunotherapy**	VEGF and angiopoietin 2		Targeting VEGF and angiopoietin 2 in combination with anti-PD-L1 antibody improved the survival of mouse breast cancer model
**Targeting epigenetic regulators**	VEGF-C via miR-128		Overexpression of miR-128 resulted in reducing the expression of VEGF-C and subsequent suppression of angiogenesis in NSCLC tumor xenograft model
**Alternative anti-angiogenic compounds**	HIF-1α, VEGF, VEGFR, PKC and endostatin via EGCG		EGCG reduced the density of tumor vessels and inhibited angiogenesis in breast and lung cancer xenograft models
**Clinical Studies**			
**Clinical Trial ID**	**Phase No.**	**Treatment**	**Condition or Disease**
**NCT03377023**	Phase I/II	Combination of Nintedanib with nivolumab and ipilimumab	Advanced or Metastatic Non-small Cell Lung Cancer
**NCT02299141**	Phase I	Nintedanib	Metastatic Non-small Cell Lung Cancer That Cannot Be Removed by Surgery and Mutations in Nintedanib-Targeted Genes
**NCT00428545**	Phase I	Combination of Bevacizumab and Bortezomib	Advanced Malignancies including breast and lung cancer
**NCT01454102**	Phase I	Combination of bevacizumab with anti-PD-1 nivolumab	Non-small Cell Lung cancer
**NCT03072992**	Phase II	Combination of curcumin and paclitaxel	Advanced and metastatic breast cancer

## Abbreviations

VEGF	Vascular endothelial growth factor
TSP	Thrombospondins
PGF	Placental growth factor
VEGFR	Vascular endothelial growth factor receptors
SCLC	Small cell lung cancer
NSCLC	Non-small cell lung cancer
TKI	Tyrosine kinase inhibitor
OS	Overall survival
PFS	Progression free survival
HIF-1	Hypoxia-inducible factor
pCR	Pathologic complete response
DFS	Disease-free survival
PDGF	Platelet-derived growth factor receptor
FLT3	Fms-related tyrosine kinase 3
PD-L1	Programmed death- ligand 1
HDAC	Histone deacetylase
SAHA	Suberanilohydroxamic acid
TSA	Trichostatin A
NP1	Neuropilin1
NP2	Neuropilin2
miRs	MicroRNAs
EGCG	Epigallocatechin-3-O-gallate
PFKFB3	Phosphofruktokinase-2/fructose-2,6-bisphosphatase 3
MCT4	Monocarboxylate transporter 4
ATP	Adenosine triphosphate
MMP	Matrix metalloproteinase
TAM	Tumor-associated macrophages
TEM	TIE2 expressing macrophages
HSP	Heparin sulfate proteoglycan
bFGF	basic fibroblast growth factor
FGFR	Fibroblast growth factor receptor
IL-6	Interleukin-6
EC	Endothelial cells
VM	Vascular mimicry
VE-cadherin	Vascular endothelial cadherin
PECAM	Platelet EC adhesion molecule
PA	Plasminogen Activator
MCT4	Monocarboxylate transporter 4
EMT	Epithelial mesenchymal transition
EGFR	Epidermal growth factor receptor
